# Submicrometer Linewidth Metrology In the Optical Microscope

**DOI:** 10.6028/jres.092.017

**Published:** 1987-06-01

**Authors:** Diana Nyyssonen, Robert D. Larrabee

**Affiliations:** CD Metrology, Inc. Germantown, MD 20874; National Bureau of Standards Gaithersburg, MD 20899

**Keywords:** critical dimension measurement, linewidth, micrometrology, microscopy, optical metrology, process-control metrology

## Abstract

The recent impetus of the semiconductor industry toward submicrometer feature sizes on integrated circuits has generated an immediate need for measurement tools and standards suitable for these features. Optical techniques have the advantages of being nondestructive and of having high throughput, but the disadvantage of using wavelengths comparable to feature size which results in complex scattered fields and image structures that are difficult to interpret. Although submicrometer optical linewidth measurement is possible for 0.3 *μ*m feature sizes, current instrumentation and linewidth standards, particularly for wafers, will have to radically improve in accuracy as well as in precision to meet the anticipated needs of the integrated circuit (IC) industry for submicrometer dimensional metrology. This paper discusses the effects of inadequate precision and accuracy on process control in IC fabrication and suggests some ways of circumventing these limitations until better instrumentation and standards become available.

## Introduction

Until relatively recently, optical linewidth measurement systems were the only practical tools for monitoring feature sizes produced by lithographic processes. With the shrinking of feature dimensions to the submicrometer level, and the concern over diffraction and wavelength limitations of optical tools, many fabrication lines jumped to scanning electron microscope (SEM) measurement tools as the panacea to all of the problems and limitations of existing optical systems. In response, new optical systems have appeared including ultraviolet and laser scanning systems. This paper and an accompanying paper on SEM systems in this issue of the *Journal of Research* [1]^1^, assess the capabilities and limitations of each of these technologies and look at how well they will be able to meet the measurement needs of present and future semiconductor processing technologies.

In the optical arena, diffraction effects due to the wavelength of light being comparable to the feature sizes of interest are the major limitation. With the use of shorter ultraviolet wavelengths (e.g., as as low as the 366 nm line of mercury), optical measurements are possible for linewidths down to about 0.3 *μ*m (Airy disk diameter is 0.45 *μ*m for f/1 optics at 366 nm). However, to go to this narrow a linewidth, it is necessary to model the effects of diffraction in the image and develop a meaningful criterion of which point on the image profile corresponds to the edge of the line. This modeling becomes increasingly difficult as the feature height becomes larger than about one-quarter wavelength and as the aspect ratio (feature height/width) approaches and becomes larger than unity. This difficulty is partly mathematical (e.g., the feature cannot be treated as planar using scalar theory and, for small linewidths and large aspect ratios, diffraction effects from adjacent edges interact). The difficulties are also partly due to the fact that the effects of diffraction become more pronounced and propagate further from the edge as the feature height increases and the geometry of the edge departs more from an ideal vertical shape. Indeed, for large aspect ratios and nonvertical walls, the very definition of “linewidth” is open to interpretation.

## Definition of Linewidth

**Figure 1:**
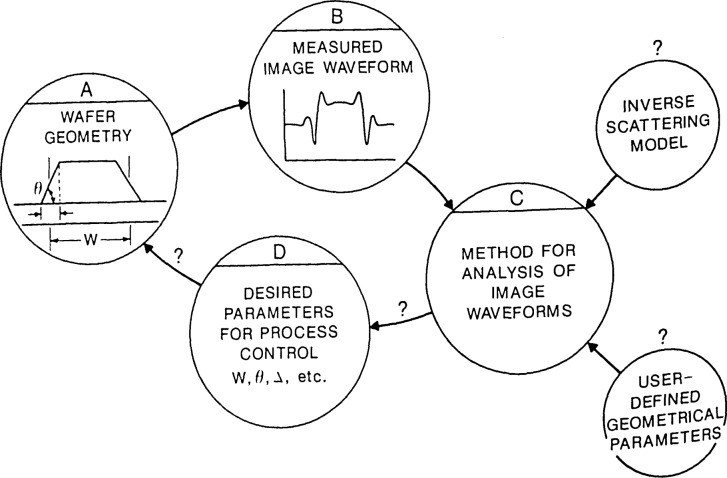
The wafer submicrometer feature-size measurement problem of relating geometry of the feature to the image waveform and developing accurate algorithms for analysis.

With linewidth standards such as the NBS photomask linewidth Standard Reference Materials, SRMs 474 and 475 [2], the fundamental limitation on the quoted uncertainty in linewidth is not due to the precision (standard deviation, *s*) of the calibration system, but rather to the definition of linewidth for sloping edges when the slope angle is not under tight control and not easily measured. The current statement of uncertainty accompanying these SRMs is based on the sum of two contributions, one from the nonreproducibility of the measurement system (approximately 0.01 *μ*m, 3*s*) and a larger contribution from a systematic error in edge detection due to the variation in edge slope which occurs during fabrication of the photomasks (see fig. 1a). This latter contribution for this standard (i.e., Δ) is estimated to be 0.05 *μ*m and is based on the fact that the NBS photomask calibration system (using transmitted light, broad spectral bandwidth peaked at 530 nm, and coherent edge detection) cannot detect the difference between a vertical edge and a θ=70° edge slope. (They both produce the same signal.) Hence, for a 150 nm thick chromium oxide/chromium layer, a 70° edge slope produces a 0.05 *μ*m “edge width.” Since it is impossible to say what point on this portion of the edge corresponds to the measured linewidth, a systematic error ±0.05 *μ*m (worst case) is assigned to the measurement.

In order to reduce this systematic error, the contribution from the uncertain edge position must be reduced. There are two possible ways of achieving this: either the photomask edge slopes must be maintained at angles closer to the vertical (where linewidth is unambiguously defined); or, if the edge slope cannot be adequately controlled, the slope angle, “edge width” or other equivalent parameter must be measured and used to characterize the edge geometry. Currently, the measured quantity of “linewidth” reveals nothing about the true geometry of the line edge in either an optical or SEM measurement. Therefore, the real concern for future technology is whether either SEM or optical technology will be able to go beyond the vague concept of “linewidth” and yield more accurate information about the true edge geometry.

## Precision and Accuracy

In metrology [3], *precision* or repeatability is defined as the spread in values associated with repeated measurements on a given sample. That is, the measurement of a given quantity will produce measurements which can be averaged to produce a mean value

$$\bar{x}=\frac{\sum_{i=1}^{n}x_{i}}{n}$$ (1)

where *x_i_* is the result of the *i*-th measurement and *n* is the total number of measurements. The *precision* or repeatability is characterized by the standard deviation

$$s=\left[\frac{\sum_{i=1}^{n}{\left(x_{i}-\bar{x}\right)}^{2}}{n-1}\right]1/2$$ (2)

These general formulas assume that *n* is large and that the errors are random and result in a Gaussian or “normal” distribution centered about the true mean. In many cases, such as length metrology, this may not be true. For example, a common source of error in dimensional measurements is misalignment of the target to be measured to the axis of the measuring instrument which varies randomly with each insertion of the target into the instrument. In this case, misalignment always causes linewidth measurements that are too large.

There is also the question of what is meant by a given standard deviation or statement of precision for a measurement system. For example, a system may be highly repeatable over a period of a few minutes but be extremely temperature dependent such that temperature fluctuations during the course of the day may produce much larger measurement variations. The first time period refers to short-term precision which is what is usually quoted by the instrument manufacturer, while the latter refers to long-term precision which is heavily dependent not only on the long term stability of the instrument but also on its environment. If the system is expected to hold calibration over periods of weeks or months without recalibration taking only control chart measurements, then the only meaningful precision statement must be based on measurements taken over that same long period of time, i.e., weeks or months.

*Accuracy*, on the other hand is a more ambiguous and elusive concept. Usually, there is some agreed-upon quantity which one is trying to measure. However, when examined in detail, this quantity and its definition frequently become fuzzy and may escape clear definition.

**Figure 2:**
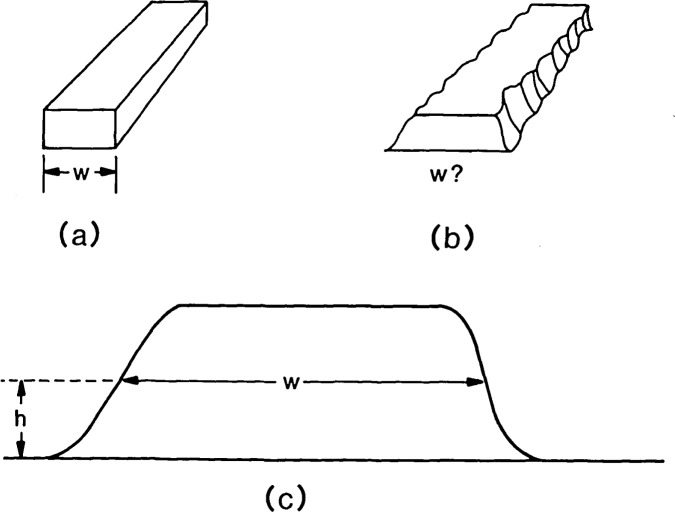
Definition of linewidth: (a) Ideal line geometry with width *W* unambiguously defined; (b) real line structure showing asymmetric, non-vertical edge geometry and edge raggedness; (c) proposed definition of linewidth as width *W* defined at some height *h* above the interface (between the patterned layer and sublayer) and averaged along some length of the line. The height *h* is selected appropriate to the application, e.g., near the interface for patterned resist.

For example, linewidth on integrated circuit (IC) features seems a clear enough idea until one begins to look at real structures. In figure 2(a), the line has an ideal structure with vertical walls and smooth edges and linewidth can be unambiguously defined as indicated by *W.* Real structures, like that shown in figure 2(b), do not have well-defined edges. They may have an asymmetric, nonvertical geometry with raggedness along their length. The only meaningful measurement on specimens with ragged edges may be an average along some specified length of the line. In different applications, the basic quantity that is desired to be measured and called linewidth may be different, e.g., the width at the bottom when either etching or doping will be the next process step or the mean width if comparisons with electrical linewidth measurements will be made.

**Figure 3:**
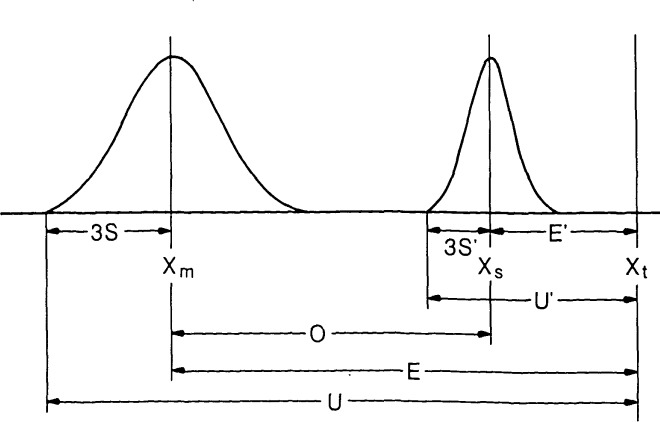
Definition of uncertainty *U* and standard deviation *s.* In this Figure: *X*_t_ is the “true” value or desired value of the measurement, *X_s_* is the value assigned to the standard with its precision given by 3*s*′ and total uncertainty *U*′, *X*_m_ is the result of measurement on another system with precision 3*s*. If the measurement offset *O* is eliminated by correction to the value of the standard *X*_s_, the uncertainty *U* associated with *X*_m_ is still at least *U*′ *+* 3*s.* Note that *X*_t_ is frequently ill-defined and that when the characteristics of the standard used to determine the offset *O* do not match those of the part to be measured, the uncertainty in *X*_m_ may actually be larger than indicated.

Therefore, a more refined definition of linewidth is needed. For instance, we may agree, as has been proposed, to measure the line structure at some distance above the interface, averaged along a 1 *μ*m length as illustrated in figure 2(c). The problem then becomes one of determining how well a given instrument can measure the agreed-upon quantity. If the system measures such a quantity with a systematic error, i.e., it always measures too large or too small, the average error or offset can be determined from measurements on a reference standard with known values. This average error is defined to be the accuracy of the measurement. The ideas of accuracy and precision can be combined [3] into what we here call the uncertainty (see fig. 3):

U=E+3s. (3)

When a measurement is given as *x* ± *U*, the desired quantity may lie anywhere in the interval defined by *±U.* If more measurements are made, they can be averaged and the precision improved in eq (2) by dividing by $\sqrt{n}$. The accuracy *E* will remain the same unless a systematic correction to the data can be determined by calibration to a reference standard. Notice that, even when a reference standard is used, *E* cannot be reduced to zero. The calibration standard has some stated accuracy and precision associated with it as illustrated in figure 3:

U′+E′+3s′. (4)

When measurements are corrected by subtracting the average difference between the known values and results of measurements on the reference standard, the total uncertainty becomes

$$U=E\prime +3\sqrt{s^{2}+{\left(s\prime \right)}^{2}}.$$ (5)

The rule here is that random errors are added in quadrature, but systematic errors must be added linearly [4]. Even when measurements are not corrected to the reference standard because the measured values lie within the stated uncertainty of the standard, the measurement system cannot be stated to have an uncertainty less than the standard to which it is compared.

Everyone wants accuracy in measurements but, unfortunately, accuracy is only achieved with expenditure of time and effort. A necessary, but not sufficient, condition for accuracy is precision (reproducibility). There are at least four main causes of imprecision in submicrometer optical metrology: 1) variations in the conditions of measurement (e.g., focus), 2) perturbing environmental conditions (e.g., vibration), 3) variations in human judgment (e.g., deciding where the feature edges are located), and 4) variations in the characteristics of the specimens being measured (e.g., thickness of features). Some of these factors can be eliminated (e.g., automation can eliminate some sources of human-induced imprecision) and some can be minimized (e.g., sources of vibration can be identified and remedial measures taken). Some of the remaining (perhaps unknown) sources of imprecision are random (e.g., noise) and thus reducible to acceptable levels by averaging repeated measurements. However, if the remaining errors are not random, (e.g., variations in the image profile resulting from contamination deposited on the surface) no amount of averaging will reduce them! Therefore, a well thought out procedure of measurement based on sound metrological principles can significantly improve precision (e.g., specifying that measurements be taken in the center of the field of view to minimize off-axis aberrations of the optical system). It is not the purpose of this paper to list all possible sources of imprecision or to recommend a universal procedure for obtaining the best precision. However, one purpose is to point out that one very important step toward accuracy is to recognize and control all known or suspected sources of imprecision.

One does not need an official standard to measure instrument precision. A typical specimen of the type to be measured (test wafers or a sample of the product) and known to be stable in time will suffice. One determines long-term precision by repeated (e.g., hourly, daily, weekly, or monthly) measurements on this type of control specimen and then applies well known quality control charting [4] or equivalent procedures to determine control limits and thereby ascertain that the measurement is under control. However, the attainment of the required degree of long-term precision does not guarantee accuracy. Given precision, there are two main sources of inaccuracy in optical submicrometer metrology; 1) lack of a generally accepted standard of comparison, and 2) improper use of standards. If suitable standards are not available, there are probably good technical reasons for their unavailability, and that reason will probably determine what can, or cannot, be done about it. The temptation is to use the best in-house control specimen as a standard. This may be acceptable as a temporary expedient if done correctly.

Accuracy may be achieved only if the instrument is sufficiently precise and if the specimens of interest exactly match the standard in all important ways except the dimension(s) being measured. However, for linewidth metrology on wafers, one usually cannot guarantee that the specimens to be measured will match the standard in feature height, in substrate properties, in edge geometry and irregularity, in complex index of refraction of the feature material, etc. Recent modeling efforts [5,6] indicate that all these things should be considered to be of prime importance. Clearly, it is inappropriate to use a thin-layer metal-on-glass photomask standard such as NBS 474 or 475 [2] to “calibrate” a system which subsequently will be used on other types of specimens (e.g., photoresist lines on silicon wafers). As mentioned above, it may be appropriate to use in-house control specimens as temporary calibration standards but, if and only if, they closely match the specimens, or range of specimens, to be measured and are known to be stable in time. Some of the material to follow in this paper is intended to be a guide to those factors that must be considered in matching such a standard to the specimens to be measured. This is important because, until accurate measurement systems and standards become available, instrument precision may be the best that can be achieved.

## Effect of Measurement Errors On Process Control

**Figure 4:**
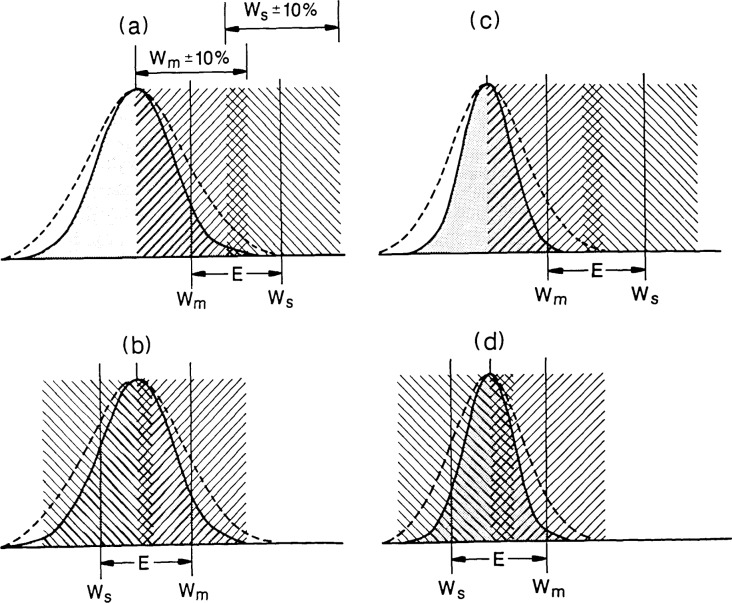
Effect of measurement errors ± *E* on acceptance and rejection of measured parts with normal distribution of measured values. Shaded area represents Gaussian distribution of measurements made on a batch of parts. Shaded 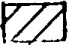 area represents tolerance window for acceptable parts as defined by measurement system. Shaded 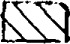 is true tolerance window defined with respect to an accurate standard. Offset is, therefore, the difference between the measured value *W*_m_ and that of the standard *W*_s_. When an accurate standard doesn’t exist, the value of *E* is unknown.

It is generally accepted that some linewidth measurements are a necessary form of process control. When accuracy and precision satisfy the gaugemakers rule, i.e., that the measurement system be 3 to 10 times better than the system that generated the part, the argument is incontrovertible. However, when the accuracy and precision of the measurement system are on the same level as the tool being monitored, the situation changes radically. Suppose, as shown in figure 4, the nominal desired linewidth *W*_s_ is 1.0 *μm* with a 10% tolerance (±0.1 *μ*m) specified. Suppose that a single measurement is made on a sampling of parts and that the resulting parts are found to have widths that are normally distributed centered 0.1 *μ*m off the nominal W_m_ with a spread of ±0.20 *μ*m (3*s*, where *s* is the standard deviation). Assume the long term precision of the measurement system is 0.1 *μ*m (3*s*) and that an unknown offset of ±0.15 *μ*m exists. The instrument precision when taken into account increases the actual spread in the dimensions of the parts (dashed curve in figure 4) as compared to the standard deviation of the measurements. An offset *E* typically occurs when the parts being measured differ in form or substance from the standard used for daily calibration of the measurement system. In figure 4a, a negative offset is shown, such that if 50% of the parts are assumed to be within tolerance, 42% of them are bad and only 8% of them meet specifications. If the part was initially overspecified, the situation may not necessarily raise alarm, if the 50% rejection rate could be tolerated.

In figure 4(b), the offset is of the same magnitude, but positive rather than negative and again, unknown to the process control engineer. If, as before, 50% of the parts are accepted based on belief in the measurements, 32% bad parts will have been accepted and 42% of good parts would have been rejected. In this case, it would have been better to accept all the parts without testing. This would result in no rejected parts (no losses) and the percentage of good parts accepted would have been higher, 59% as compared to 36%.

If the process is in tighter control so that the variation in dimensions as given by 3*s* is only 0.1 *μ*m rather than 0.2 *μ*m, the results are even worse. The offset of −0.15 *μ*m, [fig. 4(c)] results in the same acceptance rate (50%). However, none of the parts really meet spec because the unknown offset is larger than the spread in linewidths. Even if the tolerance was overspecified, the situation would not improve greatly; there would still be a large percentage of bad parts accepted. Furthermore, demanding improved precision of the measurement system by itself would not improve the situation.

If, as shown in [fig. 4(d)], the offset is in the opposite direction, tighter process control improves the situation somewhat. However, acceptance of all parts without testing would still result in a higher percentage of good parts accepted, 76% as compared to 52%, and no parts would be lost through rejection. However, since the offset is unknown, the actual situation may be any of the above and the process is clearly not in control.

The conclusion, here, is that the concept of achieving quality by using process control measurements only works when the measurement system has accuracy and precision much better than the variation in the parts being measured (the gauge-makers rule again). This above analysis leads one to the conclusion that at least half of the linewidth measurements currently made at micrometer and smaller dimensions on wafers during manufacture are probably useless if not downright damaging. It becomes obvious, therefore, that to make linewidth measurements an effective process control tool for submicrometer and future technologies, the accuracy as well as precision must be radically improved.

## Optical Linewidth Metrology

Many of the potential sources of measurement error mentioned above can be eliminated, circumvented, or minimized by the use of high quality optical and electronic systems in a suitable environment coupled with the use of sound metrological techniques of measurement and data reduction. One of the least understood and most often encountered sources of error is that associated with edge detection, that is, the location of the edge on the image profile of the feature. Ideally, as illustrated in figure 1, one would like to determine the actual geometrical shape of the edge of the feature from its measured optical image profile, decide what point on that shape should be taken as the “edge,” and then determine what point on the optical image profile corresponds to this definition of edge. The first of these steps (determination of the actual geometrical shape of the edge) from an appropriate optical signal is the most difficult, and to date, has not been treated adequately for any but the most simple cases (e.g., for photomasks). This is not an exercise of image analysis in computer programming, but a fundamental inverse scattering problem in optical imaging theory [7]. Until this problem is solved under assumptions appropriate for some real instrument, it will be impossible to accurately measure the dimensions of any thick features by optical techniques. As pointed out in the companion paper [1], there is also an analogous problem in scanning electron microscopy that must be solved before feature dimensions can be measured accurately by that technique. This is, in fact, the reason why NBS has not issued linewidth standards for anything but thin layers of metal lines on glass (i.e., photomasks). NBS is working on the problem for both the optical and scanning electron microscope cases, but the magnitude of the problem and the generality of the solution needed (e.g., applicable to a wide variety of structures, feature materials, and measuring systems) will require first the development of practical solutions, and then their application to standards.

**Figure 5:**
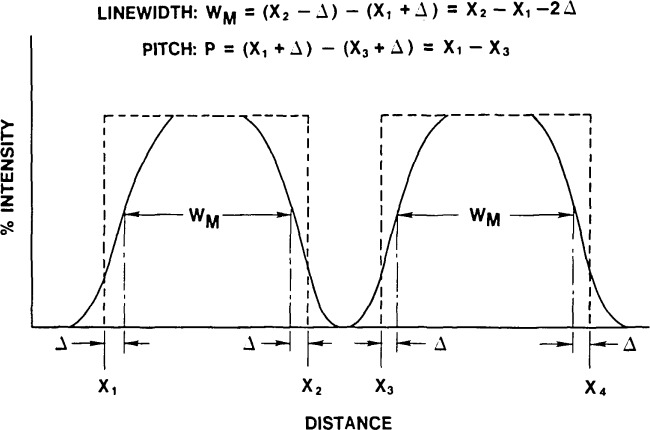
The effect of the edge- detection error (Δ) caused by use of an incorrect threshold on pitch and linewidth measurements.

Pitch measurements are not particularly sensitive to the accuracy of edge detection because, if two lines have identical geometrical edges and thus identical image profiles, the distance between them can be measured as the distance between corresponding “edges” irrespective of the edge detection criterion used (see fig. 5). For line and space widths, however, any errors in edge detection of left and right feature edges do not cancel by symmetry, but add because of asymmetry, and produce a result with twice the individual edge detection error. Therefore, use of a pitch standard for linewidth measurements will not lead to an adequate calibration for either an optical or an SEM system.

Features with heights larger than approximately 1/4 the wavelength of light (thick layer) cannot be approximated as thin layers and the image profile of such features will depend on all of the parameters mentioned above. Therefore, it is not sufficient to use a thin-layer (photomask) standard to determine the edge detection criterion for an instrument and then use this criterion for anything but similar thin layers. This is true in spite of the fact that the width of the line on the standard may be known and, perhaps, traceable to NBS. At the present time, there is no way to provide traceability of optical linewidth measurements of any feature dimensions on silicon wafers to NBS. The situation is even worse for two-dimensional features such as vias at micrometer and smaller dimensions, where the specimen cannot be taken as uniform in one dimension and both lateral dimensions must be modeled.

All of these factors combine to produce a situation where it is not possible, at present, to attain accuracy or traceability to NBS in critical dimension measurements for most features of interest to semiconductor device and circuit processing. Precision and a crude assessment of the accuracy may be attainable if the time and effort is taken to do the critical dimension measurement carefully and correctly. The first step in doing this carefully and correctly is to understand the metrologically important factors in the measurement and their reduction to practice in the instrument used for the measurement.

## Measurement of Small Feature Dimensions

In order to assess the capabilities of a linewidth measurement system, it is necessary to separately consider two parts of the system: 1) the portion of the system that acquires the signal which is usually called the image profile or waveform, and 2) the edge detection algorithm used to extract a linewidth from this signal. Even when the image profile is not digitized and stored in the system, this profile is the basic signal whose reproducibility must be considered when discussing instrument precision. Many factors effect the reproducibility of this waveform. The factors related to instrument quality have been previously discussed in the literature including coherence, aberrations, focus, alignment, vibration, etc. [8]. The basic factors to be discussed here are concerned with spectral bandwidth, mode of illumination and mode of collection of the light. The signal waveforms produced are different when the same diffraction-limited high numerical aperture (N.A.) optics are used in imaging systems differing in these respects. Therefore, the edge detection algorithm used must be tailored to the system and include its spectral bandwidth, mode of illumination, and mode of collection.

**Figure 6:**
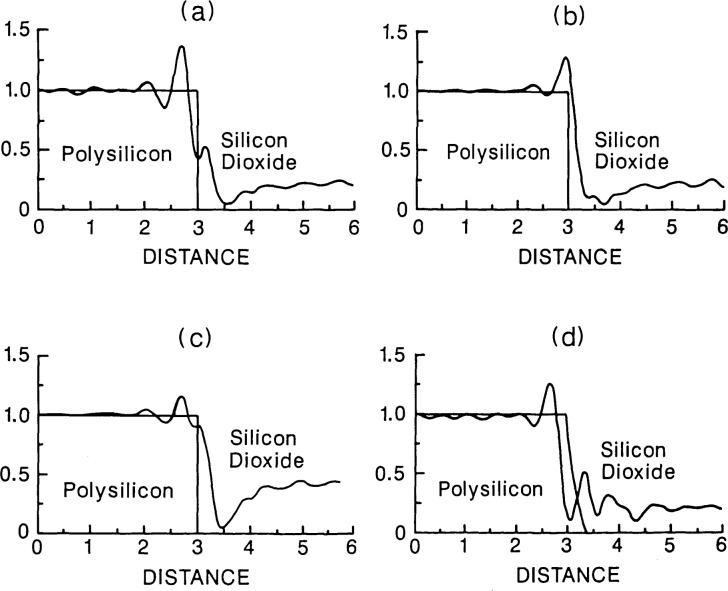
Factors which affect the image waveform: example is calculated for a 0.6 *μ*m thick polysilicon line on a 0.105 *μ*m thick oxide layer on silicon for (a) vertical edges, (b) with change in thickness of polysilicon layer to 0.65 *μ*m, (c) with change in oxide thickness to 0.125 *μ*m, and (d) with change in edge geometry. Edge geometry is shown superimposed on image profile for reference. The lines are assumed to be symmetric about their centers and only the right half is shown.

In addition, the geometrical characteristics of the sample affect this signal waveform. Some of these characteristics are illustrated in figure 6. Figure 6(a) is the image of a polysilicon line with vertical edges. In figure 6(b), the thickness of the patterned layer has changed, in (c) the thickness of the oxide sublayer has changed, and in (d) the edge geometry has changed, all resulting in changes in the image waveform. These figures were computed for the case of a narrow illumination cone angle (0.17 N.A.), λ=530 nm, and a 0.85 objective N.A. For larger illuminating cone angles and broader spectral bandwidth, these effects are still present but must be treated by integration over the appropriate cone angle and spectral bandwidth for the instrument. This integration can add considerable complexity to the task of modeling the image waveform for such instruments.

In terms of controlling the precision of the measurement system, a great deal of effort has been spent by instrument manufacturers on focus control and repeatability. With a given sample, it is very apparent that small amounts of defocus change the image waveform and, therefore, introduce an error in linewidth measurement. Similarly, small changes in the sample (e.g., thicknesses or edge geometry) also discernibly effect the image waveform and, unless taken into account by the measurement system, contribute to imprecision and loss of control. This change which results in the image waveform no longer matching that of the standard used for calibration, introduces an unknown offset or error in the measurement. These offsets vary and are estimated to be as large as 0.3 *μ*m or more on processed wafers [9].

The major cause of this offset is the use of a fixed edge detection algorithm, which does not take into account changes in the characteristics of the sample being measured. Instrument manufacturers generally leave the choice of edge detection algorithm to the user with little, if any, guidance as to what is appropriate for a given sample. The edge detection algorithms available are few, often not based on sound metrological principles, and usually not able to adapt to (or detect) changes in sample geometry, thereby turning linewidth metrology into a poorly practiced black art rather than a science!

## Instrument Design

The most important factors which influence the signal waveform are those of the instrument itself including 1) spectral bandwidth, 2) mode of illumination, and 3) mode of collection or imaging of the specimen. Although most optical microscopes are designed for use at specific wavelengths, the quality of the optics is not the primary reason for restricting spectral bandwidth. Virtually all of the materials of concern in IC manufacture are patterned layers and the restriction on spectral bandwidth is done for the same reason that a single wavelength source is desirable in ellipsometry: the waveforms of interest vary with wavelength.

**Figure 7:**
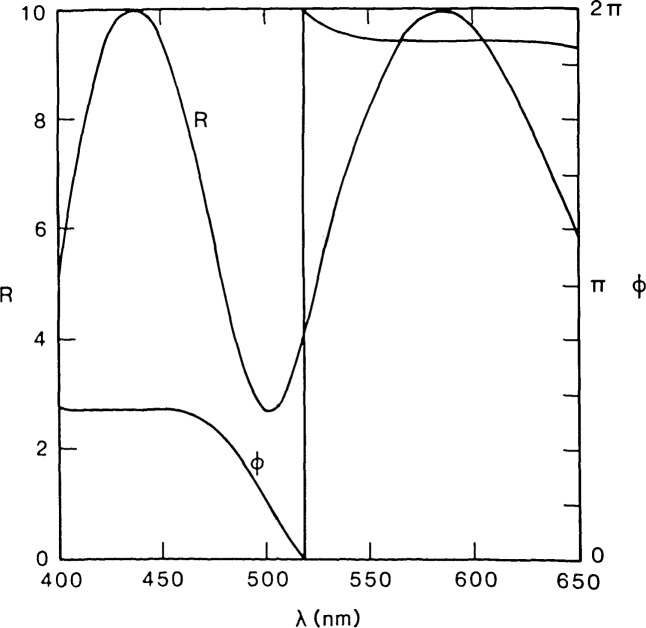
Relative reflectance *R* and phase differences ϕ for a 0.6 *μ*m thick layer of silicon dioxide on silicon calculated from the Fresnel equations for varying wavelength. Curves are normalized with respect to the *R* and ϕ parameters of the bare silicon substrate.

Figure 7 illustrates the calculated effect of wavelength on normalized reflectance (inverse of contrast) and phase step at the edge (optical path difference plus phase difference on reflection from thin films) for a 0.6 *μ*m thick patterned dielectric layer. The thicker the layer, the more rapidly the image waveform changes with wavelength. Use of broad spectral bandwidth integrates the response over the bandwidth of the system resulting in a loss of sensitivity and edge acuity.

**Figure 8:**
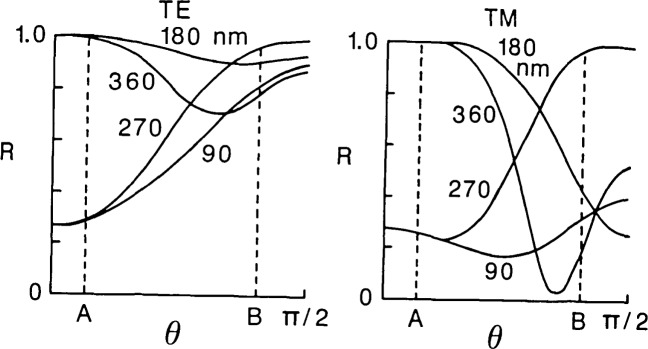
Variation of relative reflectance *R* as a function of angle of incidence θ for SiO_2_ patterned layers of varying thickness (on silicon), λ=530 nm. Dashed lines correspond to a cone angle of (A) 0.22 N.A. and (B) 0.95 N.A. Curves are shown for both transverse electric (TE) and transverse magnetic (TM) directions of polarization.

The variation of spectral response is obvious to one who has observed the rich color variations of processed wafers or has used a color chart to determine oxide thickness. The effect of angle of illumination can similarly be observed by tilting the wafer and noting the change in color with viewing angle. Wavelengths that have a high reflectance at one angle of incidence will have a lower reflectance at another angle allowing a different wavelength to determine the observed color. Therefore, it would not be surprising to see a parallel beam of laser light cause the contrast of the patterned wafer to change with variation in the angle of incidence (see fig. 8). What is perhaps less obvious, is that a focused laser beam has the same general effect to that of broad spectral bandwidth. The focused laser beam may be viewed as the sum of plane (collimated) waves corresponding to each differential element of solid angle within the cone angle of the illuminating lens [10], with each angle producing a different image waveform. Again the effect of integrating (here over the cone angle of the lens) is one of poorer edge acuity and loss of sensitivity to edge geometry resulting in a larger uncertainty in the measurement. That is, the system may produce the same signal waveform and linewidth measurement for a range of object thicknesses, edge geometry and geometrical widths. This could result in an apparent increase in precision (i.e., a decrease in the standard deviation) but, because of the insensitivity to geometry, the uncertainty in the measurement increases, and the ability of the system to detect changes affecting device performance may be lost.

**Figure 9:**
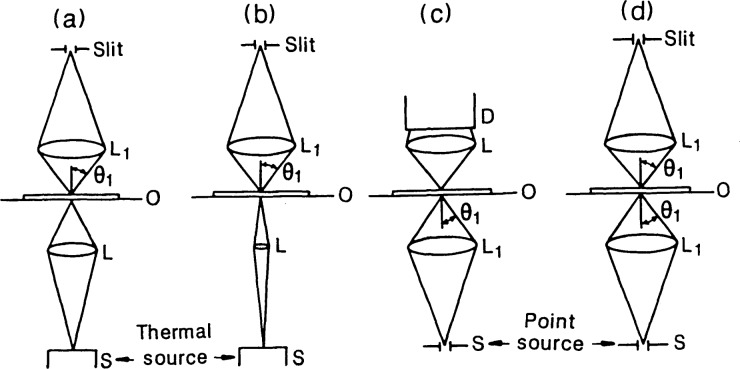
Basic optical system designs used for feature-size measurement: (a) conventional bright-field (partially coherent) with broad spectral bandwidth, (b) narrow cone-angle, bright-field (effectively coherent with single wavelength laser source), (c) focused laser beam, and (d) confocal microscopes. The systems are shown in transmission (unfolded for reflection) and in each case, the source S illuminates the line object O through a lens L. A laser source is considered to be a point source located infinitely far away from the lens L_1_ in (c) and (d). The scattered light is collected through a second lens and images onto the detector. In (a), (b) and (d) the slit at the image plane is unresolved when projected back onto the object plane. In (c), the detector D collects all of the light; it, therefore, may be placed in either the image plane, at the lens as shown, or the lens L may be eliminated altogether. Although the schematics are shown for critical illumination, Kohler illumination may be used in (a) and (b) without changing the system response.

The mode of collection of the signal energy coupled with the mode of illumination determines the resolution and coherence properties of the system which are separate from the effects discussed above. The most commonly used configurations for illumination and collection are illustrated in figure 9, i.e., bright-field, (a) and (b), focused-beam [11], (c), and confocal [12], (d), microscopes. All of these systems are partially coherent or effectively coherent imaging systems and are, therefore, sensitive to uniformity of phase as well as intensity across the field of view. Nonuniform phase is one source of asymmetry in the image profile. Accuracy of alignment and optical quality of the illuminating system, therefore, become more demanding than for a conventional microscope imaging system (less coherent) with the most stringent demands made by the single wavelength, narrow angle of incidence systems.

**Figure 10:**
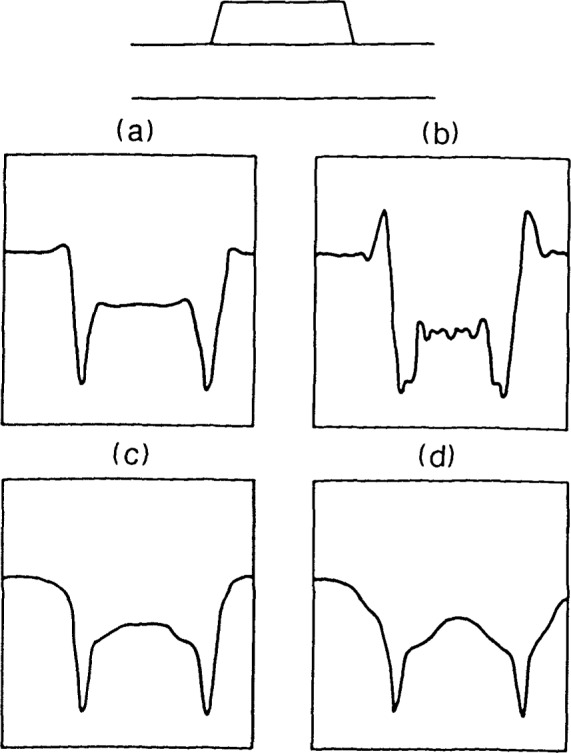
Characteristic image waveforms for line object shown at top corresponding to systems in figure 9; (a) through (d) are same as in figure 9.

For planar objects (<λ/4 thick), these four configurations of systems would be expected to produce similar image waveforms for the same numerical apertures. However, for patterned thick layer materials such as those found in IC manufacture, they do not. Characteristic image waveforms, expected for these systems are shown in figure 10. The choice of the type of image waveform becomes important when discussing edge detection algorithms for linewidth measurement. Each of these signals represents a different response to an edge discontinuity in the same material. Therefore, accurate edge detection algorithms will be different for each of these systems. To date, the only system that has been well-characterized and for which any accurate edge detection algorithms exist, is the effectively coherent (narrow illuminating cone angle) version of the bright-field microscope [fig. 10(b)] [6]. It has been shown that, as is the case with ellipsometry, it is much easier to analyze the system for single wavelength and single angle of incidence, and easier to develop accurate measurement algorithms. As in the case with ellipsometry [13], there is additional information to be gained from other angles of incidence and other wavelengths. However, integrating over a broad spectral bandwidth or a wide cone angle is not the best method to extract that additional information.

## Sensitivity, Ease of Operation, And Resulting Uncertainty In the Measurement

There are three major reasons (not necessarily advantages) for using broad spectral bandwidth and a large illuminating cone angle: 1) there is an increase in available energy and improved signal-to-noise ratio (S/N), 2) the resulting waveforms have simpler structure, and 3) there is less sensitivity to system alignment and sample differences.

Linewidth instrument manufacturers have always preferred white light sources or focused laser beam systems (lower power requirements) because of improved signal-to-noise and lower cost. In addition, manufacturing tolerances, particularly alignment of microscope parts, is less demanding. That is, integrating over a large cone angle makes the system less sensitive to both variations in sample response due to angle of incidence and errors in alignment of the optical system parts.

Recently, with the move toward automated systems and automated signal processing, the argument has been put forth that coherent (narrow angle of incidence) image waveforms are “too complex” for automated signal processing. However, the simpler waveforms (more nearly monotonic) are gained only with an accompanying loss of accuracy and sensitivity, and larger measurement uncertainties. An analogous problem exists in electron-beam lithography. With a relatively large beam diameter, the lithography system is less sensitive to an array of problems including beam stability, beam cross-section variation, vibration, positional errors, proximity effects, etc. These problems become more apparent as the beam diameter and least-countable address are reduced. Yet, no one doubts that the smaller beam produces smaller, more accurate pattern definition. It is easy to see that the less sensitive system with the larger beam diameter is coupled to a loss of accuracy and resolution. The same is true in linewidth measurement with respect to angle of incidence and spectral bandwidth.

Single wavelength, narrow cone angle, reflected-light optical systems are also more sensitive to surface contamination than systems not having these features. Although the optical imaging mechanism is different from that of an SEM system, and optical systems inherently do not deposit contamination on the surface, the problem of contamination is potentially as serious for optical systems as it is for an SEM in terms of image profile distortion. Surface contamination can result from residues of fabrication processes and airborne particles or improper handling and storage. This is one of the reasons that the use of the less sensitive transmitted-light system is recommended for measurement of photomasks [14].

## Resolution

The differences between the optical configurations shown in figure 9 with respect to resolution are small at the high N.A.’s used for linewidth measurement at micrometer and submicrometer dimensions. Bright-field and focused beam systems have the same response for the same N.A.’s and equivalent coherence parameters (ratio of condenser to objective N.A. for bright-field; ratio of collecting to illuminating N.A. for focused-beam systems [15]). Confocal microscope systems which have double the resolution at low N.A.’s show only a slight improvement in resolution at high N.A.’s [16]. There is greater potential for improvement in resolution to be gained by using shorter wavelengths. This is due to several factors: 1) the nonapplicability of small angle approximations (sin θ≃tan θ) at high N.A.’s, and 2) the loss in diffracted energy at high angles of incidence for line objects that are thick compared to λ/4. The chief advantage of the confocal system is its (sin x/x)^4^ impulse response (1-D) rather than the conventional (sin x/x)^2^ [12].

**Figure 11:**
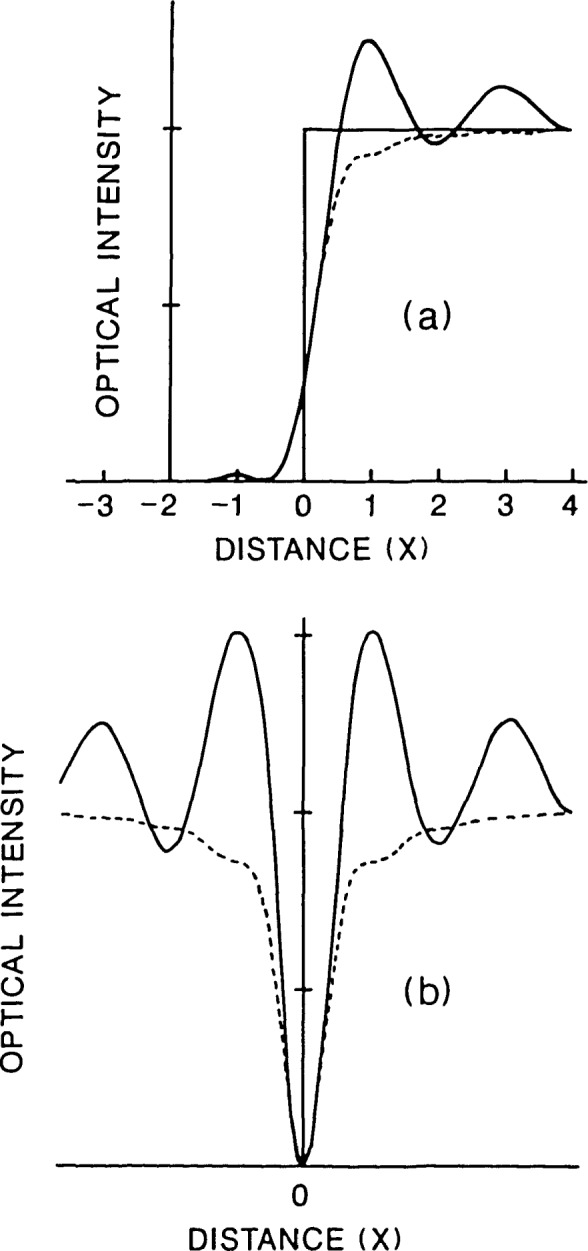
Comparison of calculated edge profiles for coherent bright-field (solid line) vs. confocal microscope (dashed line) for planar line object: (a) high contrast (opaque) with no phase discontinuity at the edge; (b) low contrast, *π*-phase discontinuity.

Comparable edge profiles are shown in figure 11 for a planar (i.e., thin) object. The fourth power function reduces the magnitude of the coherent edge ringing while still producing the minimum or dark interference band at the line edge. This type of response produces signals with less detailed structure. At the present time, however, insufficient analysis has been done to produce accurate edge detection algorithms for either focused-beam or confocal microscope systems due to the required integration over the angle of incidence for thick-layer line objects discussed above.

## Waveform Analysis—Edge Detection

Submicrometer lithography puts stringent requirements on the reproducibility of the image waveform and the accuracy of subsequent analysis of the waveform for linewidth measurement. High precision can only be achieved by controlling those factors which affect the image waveform including focus, etc. Noise also affects precision and accuracy; the two most significant sources being photon noise due to inadequate illumination levels for a given detector and vibration. As has been shown in lithography and in the SEM [1] vibration increases the apparent line dimension.

Smoothing is a frequently taken alternative to reduction of photon noise by increased source output or elimination of vibration by use of isolation systems. With “white” noise, excessive smoothing (over distances greater than the desired precision and accuracy of the measurement) results in loss of sensitivity, and changes in linewidth dimensions, the equivalent of using a larger beam size in electron-beam lithography. When noise sources such as vibration have characteristic frequencies and are not “white,” the effect of excessive smoothing is signal distortion with an accompanying loss of accuracy and precision. The best method of improvement in accuracy and precision at the nanometer level is achieved by use of brighter light sources and better vibration isolation.

## Standards

The only national or international standards currently available for linewidth measurement are photomask standards whose dimensions will shortly be extended by NBS down to 0.5 *μ*m. Methods of reducing the present 0.05 *μ*m uncertainty of the NBS photomask standard by taking into account the variable edge geometry due to process variations are currently being considered at NBS. No optical or SEM linewidth standards currently exist for features on silicon wafers. This section would not be complete without some discussion of what can be done to improve siliconwafer process control in the absence of traceability to national or international standards.

The methods of improperly applying standards to process control most often seen on IC fabrication lines include: 1) use of photomask standards for calibration of systems used to measure wafers, 2) measuring a single in-house specimen in an SEM by conventional techniques and, assuming that the results are representative of a given process step, subsequently adjusting all measurements (by addition of a “fudge factor”), or 3) measuring a single in-house standard in cross section in an SEM and similarly adjusting subsequent measurements. Each of these methods will introduce some level of variable and unknown offsets in subsequent product measurements.

**Figure 12:**
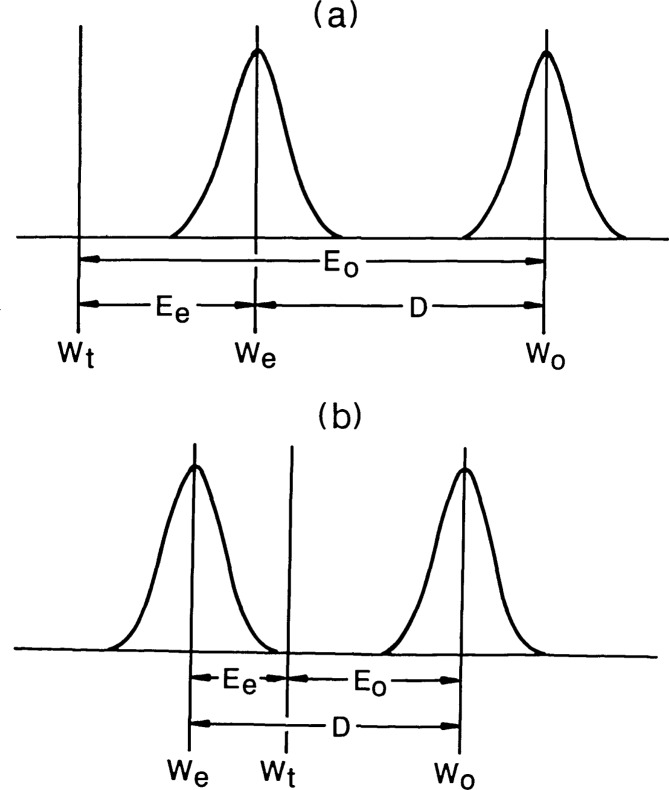
Relationship of optical (*E*_o_) and SEM (*E*_e_) measurement errors when offsets are (a) of the same sign, and (b) of the opposite sign. *W*_t_ is the desired (user-defined) linewidth; *W*_e_ the mean linewidth as measured in the SEM; and *W*_o_ the corresponding linewidth measured optically.

The worst method is the use of a photomask standard for other than pitch or line scale calibration. In addition to poor signal (or visibility) when viewed in reflected light, the chief problem is that the image profile (except in rare cases) does not match that of the wafer being measured. Therefore, any edge detection threshold or other criterion based on the certified photomask linewidths is guaranteed to be in error by an unknown amount. These errors may be as large as 0.5 *μ*m. The second choice, the use of an in-house standard measured by conventional SEM techniques also has problems. It is usually assumed that, regardless of the respective measurement and edge detection techniques used, all of the difference between the optical and SEM measurements is due to error in the optical measurement. This is an unwarranted assumption which is discussed in the accompanying paper [1]. Some error is associated with both the SEM measurement *W*_e_ and the optical measurement *W*_o_. At the present time, there is no technically sound way of apportioning the difference between SEM and optical measurement errors. It is also possible as illustrated in figure 12 that both are in error in the same direction so that the error in the optical measurement is larger (or smaller) than the measured difference, *D.*

Measurements using SEM-viewed cross sections of the lines on product wafers, while likely to reduce some of the SEM error (principally that due to interaction with the substrate and shadowing effects), do not eliminate the SEM contribution entirely and again leave the process control engineer unable to assess the true magnitude of the optical and SEM errors. Thus, if the SEM measurement is assumed arbitrarily to be accurate, some unknown offset will still be present.

In addition, all of these methods have another problem in common resulting from use of a single product sample. Both SEM and optical measurements (to different degrees) are sensitive to characteristics of the specimen such as layer thickness, edge geometry, and contamination. These variables cause changes in the image waveform resulting in a variable error or offset whenever a fixed threshold (or other edge detection criteria) is used which does not take into account changes in the material or geometry. Currently, SEM and optical edge detection criteria used are unable to adapt to changes in the image profile by appropriate corrections to the edge detection criteria used.

Thus, the examples shown in figure 4 represent actual situations which might arise in process control situations given the present state-of-the-art measurement systems and standards. There are, however, several things that the process control engineer can do to improve the situation. First, the measurement system must be under the best control possible and its long-term precision established by accepted control chart techniques. The system should be calibrated to a pitch or magnification standard. This standard need not match the material characteristics of the product to be measured since pitch or line-scale is not sensitive to edge detection errors as long as the line geometry is symmetric. Next, a test pattern or sample, characteristic of the product, can be used to determine precision for the range of linewidths of interest. This de facto product standard should be measured initially to form a data base and then repeated measurements made over many days or longer to establish the long-term reproducibility [4].

Once satisfactory precision is established, there are two remaining concerns that must be addressed: sensitivity of the instrument to changes in geometry of the lines that might effect product performance especially at submicrometer dimensions, and the relationship of the measured linewidths to product performance to establish an acceptable window for process control since the measured linewidths (because of unknown offsets) cannot be assumed to relate directly to engineering tolerances.

Sensitivity of the measurement system can be determined by examining good and bad product by other methods such as ellipsometry, profilometry, and SEM inspection in cross-section (after coating or other means to eliminate charging). For example, do two resist lines with different edge slopes, (which, as determined from SEM inspection, should show differing line widths) actually show differences in linewidth in the optical measurement system? and, are such differences proportional to the differences seen in the SEM? Since neither system can be assumed to be accurate per se, the only concern here is whether the measurement system has the sensitivity to distinguish good from bad product. This should be determined by correlating data from samples of the product showing thickness and edge geometry differences representative of what is expected in a production run.

The most difficult step is establishing the window of acceptable linewidths for a given process step and measurement instrument. The idea is to correlate measured linewidths on both good and bad product (which has been determined to have failed because linewidths are out of specification) with some performance characteristic. The innovative techniques necessary here are based on knowledge of the particular device and fabrication process. For example, where linewidth of the fabricated feature can be correlated to the operational speed of the device, electrical data on the completed devices can be used to define an acceptable range of measured linewidths. Similarly, for diffusion lines, electrical data on linewidth test patterns can be correlated with optically measured linewidths. Establishing an acceptable window for an arbitrary resist patterning step in the fabrication process presents the most complex problem. The optical linewidth measurement system is very sensitive to thickness and edge geometry changes in thick dielectric layers such as resist. The desired bottom width of the resist line is one of the most difficult to measure and thus to correlate with device performance. The acceptability of the resist profile must be determined from the acceptability of the resulting patterned layer with a window allowed for variability in the patterning process.

Although the approach described above is more demanding, it is likely to yield more satisfactory results than blind faith in the linewidth values produced by any one state-of-the-art optical or SEM linewidth measurement system when the gaugemakers rule is not met by the measurement system. If the measurement results at some stage of processing can be shown to be a valid predictor of yield, then the need for accuracy is somewhat circumvented. This approach requires the continued processing of measured specimens, and the tracking of specific specimens as they are subsequently processed and ultimately tested. However, this approach is not a substitute for accuracy because its success depends on unknown and, perhaps, uncontrolled factors besides the measured parameter(s) that affect yield. These factors lower the correlation, diminish the value of the critical dimension measurement and, if they get out of control, can dominate the yield and destroy the previously determined correlation. It is, however, something that should be done even after accuracy is achieved to validate the importance and justify the cost of the critical-dimension measurement in question.

A major problem still remains, it does little good to generate a standard with a very small uncertainty if the measurement system does not satisfy the gauge-makers rule: the accuracy and precision associated with *both* the standard and the measurement system must be 3 to 10 times better than the variations produced by the lithography tool which generated the wafer for the most effective process control. Better measurement tools and standards are, therefore, needed for submicrometer lithography.

## Alternative Linewidth Measurement Techniques

There are several alternative linewidth measuring techniques that have been suggested and, in some cases implemented, that overcome one or more of the disadvantages of the optical imaging techniques discussed above. Some, like the scanning electron microscope [1], use a different form of “illumination.” Others, like the scanning tunneling microscope [17] directly probe the feature surface topography and produce a profile. Still others, like electrical test patterns [18], do not produce images or profiles, but directly measure an average linewidth.

Although the SEM potentially has better resolution in terms of beam characteristics, the complex interaction of the electron beam with the specimen [19] currently limits the accuracy and precision available in feature-size measurement. This and other problems associated with the use of SEMs for linewidth measurement are discussed in the accompanying paper by Postek and Joy [1].

Electrical techniques based on test patterns [18] have the advantage of simplicity for both the measurement system and interpretation of the data. However, the test patterns require significant area on the integrated circuit and it is usually not possible to measure the actual lines of interest in the circuit. In addition, only conductive lines can be measured. However, the measurement is self calibrating, fast, simple to understand, and implementable with standard testing hardware. Clearly, the electrical test pattern approach has something to offer and will find its niche in semiconductor processing. Newer electrical techniques such as profiling by use of the tunnel effect [17], have potential in some applications and, at the present time, are being researched for dimensional and other applications.

Optical approaches inherently have some distinct advantages over alternatives (e.g., they are nondestructive and applicable to all materials regardless of their electrical conductivity). Because of this, there is a continual search for new ways to exploit optics to circumvent or eliminate the problems created by the relatively long wavelength of visible light. Use of shorter wavelengths is based on sound principles, but is currently limited by the availability of good quality optical elements at the shorter ultraviolet and soft x-ray wavelengths [20]. Scanning aperture (or near-field microscopy) [21,22] appears to have the advantage of circumventing the diffraction limitations of dry optics, thereby, providing greater resolution over conventional microscopy. These systems are difficult to implement and not well understood, nor have they been analyzed sufficiently for use in metrological applications.

A number of modifications of more conventional optical microscopy have been proposed (e.g., confocal microscopy [12] and phase-measuring systems [16]) but, these techniques do not circumvent diffraction effects and, presently have no metrologically sound criteria of edge detection. The authors’ believe that, as is the case with optical versus electron-beam lithography, the inherent advantages of optics will assure its niche in submicrometer critical dimension metrology for some time to come. However, that niche can only be filled by methods that are backed by sound theoretical analysis, predictions that agree with experiment, and meaningful edge detection criteria. Current research has shown that single wavelength, narrow angle of incidence microscopy is extremely sensitive to edge geometry and that, through inverse scattering, the possibility exists of extracting line geometry parameters from an optical signal. However, the development of such schemes and their analysis will take an investment in time and resources, and that is unfortunate because the rapid progress to submicrometer dimensions made by the semiconductor industry in recent years has led to needs for submicrometer metrology today. Perhaps this is the price that must be paid by an industry that tends to take metrology for granted and, in the past, has not supported metrological research and development to the extent needed to meet its future demands.

## Conclusions

Accurate measurement of submicrometer feature sizes on integrated circuits is a problem of primary importance to the semiconductor industry and one that is not likely to have an effective and efficient solution in the near future. Although optical techniques offer the advantages of nondestructive testing and relative simplicity of use coupled with high throughput, they presently are incapable of the needed precision (reproducibility), and accuracy for any but the simplest of specimens (i.e., photomasks). Suitable optical systems and associated edge-detection criteria will be developed and applied to integrated-circuit features. But until then there will be no acceptable linewidth standards for silicon wafers, and there will be no universally accepted accuracy in linewidth measurements on these wafers. With the development of suitable edge-detection criteria and the use of ultraviolet (or shorter) wavelengths, most of the submicrometer linewidth region above 0.3 *μ*m may be measurable optically. However, for the present, the semiconductor industry will, of necessity, have to use in-house standards for instrument set-up, maintenance, and quality control to gain reproducibility. The best that can be done under such circumstances is a crude assessment of accuracy based on the most accurate alternative measurements available. This unfortunate situation is due, in part, to the rapid progress of the industry in achieving ever smaller feature sizes—that progress has been faster than the developments in dimensional metrology needed to keep pace with it.

## Footnotes

1 Figures in brackets indicate literature references.
